# Radiolabeled Somatostatin Analogues Therapy in Advanced Neuroendocrine Tumors: A Single Centre Experience

**DOI:** 10.1155/2012/320198

**Published:** 2012-08-09

**Authors:** A. Filice, A. Fraternali, A. Frasoldati, M. Asti, E. Grassi, L. Massi, M. Sollini, A. Froio, P. A. Erba, A. Versari

**Affiliations:** ^1^Department of Nuclear Medicine, Azienda Ospedaliera Santa Maria Nuova, IRCCS Reggio Emilia, Via Risorgimento 80, 42100 Reggio Emilia, Italy; ^2^Department of Endocrinology, Azienda Ospedaliera Santa Maria Nuova, IRCCS Reggio Emilia, Via Risorgimento 80, 42100 Reggio Emilia, Italy; ^3^Department of Medical Physics, Azienda Ospedaliera Santa Maria Nuova, IRCCS Reggio Emilia, Via Risorgimento 80, 42100 Reggio Emilia, Italy; ^4^Nuclear Medicine Unit, University of Pisa, Via Roma 55, 56125 Pisa, Italy

## Abstract

The aim of this study was to assess the efficacy of PRRT in patients with advanced neuroendocrine tumors (NETs). *Patients and Methods*. From January 2007 to August 2011, we enrolled 65 patients (m/f 38/27; mean age 65 years, range 33–83) with advanced NETs having enhanced SSTR expression, treated with PRRT. The enhanced expression of SSTR was assessed using ^68^Ga-DOTATOC/DOTATATE PET/CT. Among all the enrolled patients, 6 of them were excluded from the present analysis since they voluntarily interrupted treatment. Mean activity/cycle of 2.6 GBq (^90^Y-DOTATOC/DOTATATE) or 6.0 GBq (^177^Lu-DOTATOC/DOTATATE) was administrated intravenously (max 9 cycles). *Results*. Complete response (CR) was found in 1/59 (2%) patients, partial remission (PR) in 24/59 (40.5%) patients, stable disease (SD) in 24/59 (40.5%), and progression (PD) in 10/59 (17%) patients. The overall tumor response rate (CR + PR) was 42.5%. In 40.5% of patients, the disease could be stabilized. Overall, 49 out of 59 patients had no tumor progression (83%). Twelve patients out of 59 (20%) had grade 2-3 hematological side effects including anemia, thrombocytopenia, and leukopenia. Long-term nephrotoxicity was observed in 3 patients (2 moderate, 1 severe). *Conclusions*. PRRT is a promising perspective for patients with advanced NETs.

## 1. Introduction

Neuroendocrine tumors (NETs) are considered a class of rare neoplasms accounting <5% of all tumors. However, diagnosis of NETs has increased substantially over the last decades and prevalence is now greater than that of any other upper gastrointestinal tumor [1]. These tumors originate from dispersed neuroendocrine cells, distributed almost ubiquitously in the body [2], and occur in 5/100,000 people per year [1]. 

The most frequent sites of NETs are gastroenteropancreatic tract (GEP NETs), followed by lungs; less frequently skin, adrenal glands, thyroid, and genital tracts may be affected. Different nomenclature systems and classifications have been used for NETs. 

Current pathological staging and grading differ between Europe and USA; however, both classification systems are centered on the primary site of the tumor and histological grade. In Europe, the Ki-67 proliferative index is used to differentiate tumors of low (<2%), intermediate (2–20%) and high (>20%) grade, whereas in the USA, tumors are graded as “well-” and “poorly-” differentiated where “well” equates to low-intermediate grade and “poorly” equates to high-grade tumors [3, 4]. 

Up to 80% of GEP NETs express somatostatin receptors (SSTR2 and SSTR5 primarily). Therefore, somatostatin analogues have been used for both diagnosis and treatment of NETs. ^111^In-labeled SST-analogues SPECT and ^68^Ga SST-analogues PET/CT represent an accurate methods for NETs diagnosis peptide radioreceptor therapy (PRRT) indication and patients management [5–8]. 

 When beta-emitters isotopes as ^90^Y (T_1/2_ of 2.67 days, maximum range of tissue irradiation of 12 mm) or ^177^Lu (T_1/2_ of 6.73 days, maximum range of irradiation of 1.5 mm) are used to label SST-analogues linked to a chelator, PRRT may be performed. After the i.v. injection, the radiopharmaceutical will distribute in the body, selectively bind to SSTRs, and actively be taken up by the cells through a process called receptor-ligand internalization [9, 10]. The internalization will ultimately lead to a selective accumulation of radioactivity in the tumor, thus determining cell death. The majority of clinical trials data available is from non-randomized retrospective case series. Due to variation in patients selection, dosing, scheduling, and total number of treatments it can be challenging to draw firm conclusions from the literature. However, it seems to be a benefit for selected patients with response rates in the range of 40% [11–14].

Here we present the results of a phase II study designed to treat disseminated or nonoperable NETs patients with PRRT. Patients demonstrated enhanced SSTR expression at PET/CT with ^68^Ga-peptide (DOTATOC/DOTATATE). 

## 2. Materials and Methods

### 2.1. Study Design

This was a prospective nonrandomized single-arm clinical trial performed at the Department of Nuclear Medicine, Santa Maria Nuova Hospital, Reggio Emilia (Italy). All patients with advanced, progressive NET fulfilling the study inclusion criteria were first evaluated with ^68^Ga-peptide PET/CT followed by ^111^In-peptide dosimetric evaluation to determine both the presence of SSTR expression as a target for the following treatment and eligibility to PRRT, that is in presence of provisional adsorbed doses: (a) >10 Gy to tumor, (b) <10 Gy to the kidneys, (c) <6 Gy for the liver, (d) <1.5 Gy for red marrow, (e) <3 Gy for lung, and (f) <8 Gy for whole body. In case of ^177^Lu-PRRT (^177^Lu-DOTATOC/DOTATATE), dosimetric evaluation was performed acquiring images during the first cycle of therapy. A fractionated treatment protocol was followed with the intravenous administration of an average activity of 2.6 GBq/cycle for ^90^Y-PRRT and 6.0 GBq/cycle for ^177^Lu-PRRT, respectively, with an interval of about 2 months. 

Toxicity and tolerability were recorded through all the study and for additionally 6 months after the study completion. Serial follow-up ^68^Ga-peptide PET/CT imaging was repeated after each PRRT cycle during the first part of the study as required by our ethic committee. The clinical trial was subsequently amended and the number of PET/CT examinations reduced to baseline, intermediate (after 2-3 PRRT cycles), and end-treatment (3–6 months after the last PRRT) scans. In order to homogenize data analysis, treatment response was assessed comparing PET/CT studies performed at baseline and at the end of treatment as well as patient's clinical response. The intermediate PET/CT evaluation was used only to assess the early progressive disease (PD).

The study was conducted in accordance with International Conference on Harmonization Good Clinical Practice guidelines, the Declaration of Helsinki and it was approved by local and national authorities (EudraCT numbers 2006-000897-65 and 2008-000983-17). 

### 2.2. Patients

From January 2007 to August 2011, we enrolled 65 patients (38 men and 27 females; mean age = 65 years, range 33–83). All patients presented progressive disease and fulfilled the following inclusion/exclusion criteria.

#### 2.2.1. Inclusion Criteria

The inclusion criteria were as follows:

- Age > 18 years;
- histological confirmation of NET; inoperable or metastatic disease;
- presence of at least one measurable lesion;
- positive 68Ga-peptide PET/CT defined as radiopharmaceutical uptake in tumor and/or metastasis higher than liver, evaluated within 3 months before PRRT (qualitative analysis);
- adequate hematological parameter: hemoglobin level (Hb) ≥10 g/dL; leucocytes (WBC) ≥ 2.5 × 10^3^/mL; platelets (PLT) ≥ 100 × 10^3^/mL;
- adequate liver and renal function: bilirubin levels <2.5 mg/dL; creatinine levels <2 mg/dL;
- ECOG performance status <2;
- Signed informed consent;
- discontinuation of cold SST-analogues treatment at least 4 weeks before PRRT;
- Life expectancy of at least 6 months.

#### 2.2.2. Exclusion Criteria

The exclusion criteria were as follows:

- other treatment (such as chemotherapy or radiotherapy) or participation in any investigational drug trial within 1 month of PRRT and for the following 2 months;
- Pregnancy or lactation;
- Bone marrow involvement >25%;
- other concomitant tumors, except “in situ” basal cell carcinoma and tumors of the uterine cervix treated with radical surgery.

Additionally, before each PRRT cycle the following parameters should be maintained: Hb ≥ 10 g/dL, WBC ≥ 2.5 × 10^3^/mL; PLT ≥ 100 × 10^3^/mL, creatinine levels <2 mg/dL; bilirubin levels <2.5 mg/dL. 

The final analysis was based on a total of 59 patients (m/f 33/26) since 6 patients (2 with GI tumor, 1 with carcinoid tumor of the lung, and 3 with pancreatic tumor) voluntarily interrupted the treatment. Tumor was localized in the gastrointestinal tract in 19/59 cases (32%), followed by pancreas in 16/59 cases (27%) and lung in 13/59 cases (22%). In 11/59 patients (19%), the origin was unknown.

All patients at enrollment had metastatic (stage IV) PD (Table 1). Histopathological findings including grading were not reported for patients since histological diagnosis was performed in different centers thus features were reported in different not comparable modalities. Previous treatments are reported in Table 2. Diabetes was present in 11/59 cases and 16/59 patients suffered from blood hypertension. Additionally, 9/59 had previous tumors (3/9 prostate cancers, 3/9 breast cancers, 2/9 large-bowel cancers and 1/59 stomach cancer) with a minimal time of free disease of 5 years. Main baseline clinical signs and symptoms were diarrhea (18/59), pain (12/59), weight loss (7/59), flush (5/59), cough (4/59), constipation (3/59), nausea (2/59), and carcinoid syndrome (1/59). Additionally, 32/59 patients presented at enrolment a variable grade of asthenia. Twenty-seven patients were asymptomatic at baseline. Serum baseline CgA levels were normal in 19/59 patients.

### 2.3. Radiopharmaceuticals Preparation

^111^In-, ^90^Y-, and ^177^Lu-peptide (DOTATOC or DOTATATE) were synthesized by following internal protocol [15]. Every preparation was obtained by carrying out the following steps: (a) a 3 mL syringe was filled with a 1 mL solution containing 30 *μ*g of sodium ascorbate and an amount of a 4 mg/mL peptide solution proportional to the ^90^Y-, and ^177^Lu- or ^111^In- activity in order to achieve a radiolabeling specific activity of 106 MBq/nmol, 48 MBq/nmol, and 6 MBq/nmol, respectively (b) this solution was added to a 3 mL Schott vial containing an activity ranging between 7.4 to 30 GBq of ^90^Y chloride solution, between 15 to 60 GBq of ^177^Lu chloride solution or between 222 to 444 MBq of ^111^In- chloride solution (Perkin Elmer, Boston, MA, United States) in 0.05 M hydrochloric acid obtaining a 4.6 pH solution; (c) the Schott vial was heated for 30 minutes at 90°C in a heating block; (d) a 5 *μ*L aliquot of the solution was withdrawn for carrying out the quality controls by using solid phase extraction or chromatographic methods [16]; (e) only for ^90^Y and ^177^Lu-peptide: the preparation was transferred to a bigger vial containing 0.5 mL of 1 mM DTPA solution and diluted with 20 mL of 0.9% sodium chloride solution [17]; (f) single doses for the patients were obtained by fractioning the mother solution in vials containing 2 mL of an ascorbic acid/sodium ascorbate buffer solution in order to decrease the effects of radiolysis. The radiochemical purity of the ^111^In-, ^177^Lu-, and ^90^Y-peptide preparations was always >99.8%. 

The radiolabeling of ^68^Ga-peptide was performed by means of a modular lab synthesizer (Eckert & Ziegler, Berlin, Germany) as already described [18]. Briefly, the fraction of about 2 mL of the ^68^Ge/^68^Ga-generator eluate containing about 80% of the ^68^Ga activity in 0.1 M hydrochloric acid was selected and directed to a reactor vial containing a 20 *μ*L of peptide solution (1 mg/mL) and 200 *μ*L of a 1.5 M sodium formate solution or 140 *μ*L of a 1.5 M sodium acetate solution in order to obtain a pH ranging between 3.2 and 3.5. The mixture was heated at 100°C for 5 minutes and, then, passed through a light C-18 cartridge. ^68^Ga-peptide was eluted with 0.5–1 mL of a 50% ethanol solution and diluted with 8 mL of 0.9% sodium chloride solution. The synthesis was carried out in 14 minutes with a mean yield of 63 ± 3% (not corrected for decay). Quality controls were performed by chromatographic methods as already described, obtaining a radiochemical purity always >95% [19]. 

#### 2.3.1. Pretherapeutic Somatostatin Receptor Imaging

Pre-therapeutic imaging was performed by ^68^Ga-peptide PET/CT. For this study, PET/CT scans were acquired on a GE Discovery at 60 min after injection of about 120 MBq of ^68^Ga-peptide. Seven or eight bed positions with 5 slices overlap were acquired for 4 min emission time in 3D. The CT-exposure factors for all examinations were 120 kVp and 80 mA in 0.8 seconds. PET images were reconstructed using CT-attenuation correction (OSEM). All studies were visually and semiquantitatively assessed. SUV calculations were performed on a Xeleris workstation. Mean and maximum SUV (activity concentration corrected for patient weight and total injected dose) was determined in all lesions and recorded. 

#### 2.3.2. Selection of Patients Eligible for PRRT

^68^Ga-peptide PET/CT was considered positive in patients who showed uptake in the tumor lesions at least two-times higher than the liver; thus they were considered eligible for PRRT and, therefore, admitted to dosimetric evaluation. 

### 2.4. Dosimetry

Planar imaging was initially performed after the i.v. injection of 185 MBq of ^111^In-peptide with a dual-head gamma camera (Genesys, Philips, The Netherlands) using parallel-hole, medium-energy, general-purpose collimators. The windows were centered over both ^111^In- photon peaks (247 and 172 keV with a window width of 20%), whereas scatter fraction was evaluated at 140 keV (width 20%). 

In all the patients, whole-body scan and, in selected cases, spot images of the abdomen were obtained after 1, 4, 20, 48, and 72 hours for control of biodistribution. To determine blood clearance, we drew blood samples at 30 and 60 minutes and at 4, 20, and 48 hours after injection. Radioactivity in blood was measured with a HPGe spectrometer (DSPEC jr 2.0—Ortec). For dosimetric calculations, regions of interest were drawn manually on the whole-body scans from anterior and posterior projections and ULMDOS software (University of Ulm, Germany) was used. Background regions were placed on the abdomen or on the thigh for background correction. Scans were corrected for background, self-absorption, patient thickness attenuation, and organ overlapping. Whole-body activity acquired immediately after injection was defined as 100% of the injected activity. Data were expressed as percentage injected activity as a function of time. The resulting time-activity points were fitted to a monoexponential or multiexponential curve for whole-body, kidneys, liver, spleen, and red marrow to calculate residence time. Patient-specific organ masses were also considered. The estimated doses delivered to critical organs and to the tumor were obtained by the software OLINDA/EXM [20]. The activity in blood was fitted to a biexponential curve to determine the residence time in blood. The dose to the red marrow was calculated from the residence time in blood, assuming no specific uptake, a uniform distribution of activity, and clearance from red marrow equal to that from blood. A correction factor of 1 was used as described by Cremonesi et al. [21].

In case of ^177^Lu-PRRT the dosimetric evaluation was performed acquiring images during the first cycle of therapy, thanks to the low gamma emission of this isotope.

### 2.5. Therapy (Administration Protocol)

A fractionated treatment protocol was followed with the intravenous administration of an average activity of 2.6 GBq and 6.0 GBq per cycle for ^90^Y-PRRT and for ^177^Lu-PRRT, respectively, with an interval of about 2 months. For each cycle, patients were hospitalized for 3 days in accordance with local requirements. Thirty minutes before administration of the radiopeptide 2 L of amino acid solution of Hartmann-Hepa 8 (Ringer's Lactate Hartmann, Proteinsteril Hepa 8%, Mg 5-sulfat) were infused, which were continued up to 3 hours after injection to inhibit tubular reabsorption of the radioactive tracer. Repeated treatments were performed in case of response and significant improvement in symptoms and quality of life, except in cases of renal toxicity and rejection by the patient for further treatment within 3 months. Additional cycles were suspended in case of PD. 

### 2.6. Biodistribution of the Radiotracer

In order to evaluate the biodistribution of therapeutic activity, after each treatment, planar imaging was performed with a dual head SPECT gamma camera (Genesys, Philips, The Netherlands) or with a dual-head SPECT/CT gamma camera (Symbia-T, Siemens, Germany) using parallel-hole, medium-energy, general-purpose collimators. The windows were centered over ^177^Lu-PRRT photon peaks (208 keV and 110 keV width 20% in both cases; scatter window at 160 keV) in case of treatment with ^177^Lu-PRRT; while at 170 keV (20%) and 80 keV (55%) in case of treatment with ^90^Y-PRRT, as Bremsstrahlung planar scan. Whole-body scans (acquisition time: 25 minutes) and spot images (acquisition time: 10 minutes) were obtained.

### 2.7. Assessment of Clinical Benefit and Evaluation of PRRT Response

Clinical benefit was assessed comparing baseline clinical conditions with end-treatment parameters. In the clinical benefit evaluation the worsening of clinical conditions (i.e., appearance of new sign(s)/symptom(s)) were considered as PD. Indeed any significant variations in baseline clinical conditions was defined as stable disease (SD). Clinical benefit was defined as non-PD/SD. For the follow-up blood tests were evaluated, as described in the clinical protocol, repeated before and after each treatment cycle and every two weeks. Blood tests included hematological parameters, liver and renal function. Baseline and end-treatment serum CgA values were compared and the trend was defined as increased, stable (variation over time ≤10%) or decreased. All patients were followed for an additional 6 months after the last radiopharmaceutical administration. Acute and long-term adverse events were graded according to the Common Terminology Criteria for Adverse Events, version 3.0 of the National Cancer Institute [22]. To assess response to treatment PET/CT studies performed at baseline and at the end of treatment were considered. 

Treatment responses assessed by PET/CT scan were defined as follows:

- complete response (CR): disappearance of radiopharmaceutical uptake in all detectable lesions;
- partial response (PR): reduction of radiopharmaceutical uptake (>50%) in all detectable lesions in absence of appearance of new lesion(s);
- stable disease (SD): no variation or reduction of radiopharmaceutical uptake (<50%) in some detectable lesions in absence of appearance of new lesion(s);
- progressive disease (PD): increase >25% of radiopharmaceutical uptake in one or more lesions or appearance of new lesions and/or >10% increasing of tumor marker.

In this series of patients, we did not assess treatment response based on the size of lesions using the CT component of PET/CT images or CT scan, but as described above we evaluate only the functional response.

### 2.8. Statistical Analysis

All values are expressed as median and range, as customary for nonparametric data.Correlation analysis was performed using the Mann-Whitney test.

## 3. Results

^68^Ga-DOTATOC/DOTATATE PET/CT was performed in all the patients to evaluate the eligibility. Baseline ^68^Ga-DOTATOC/DOTATATE PET/CT demonstrated at least one site of radiopharmaceutical uptake. 

**Figure 1:**
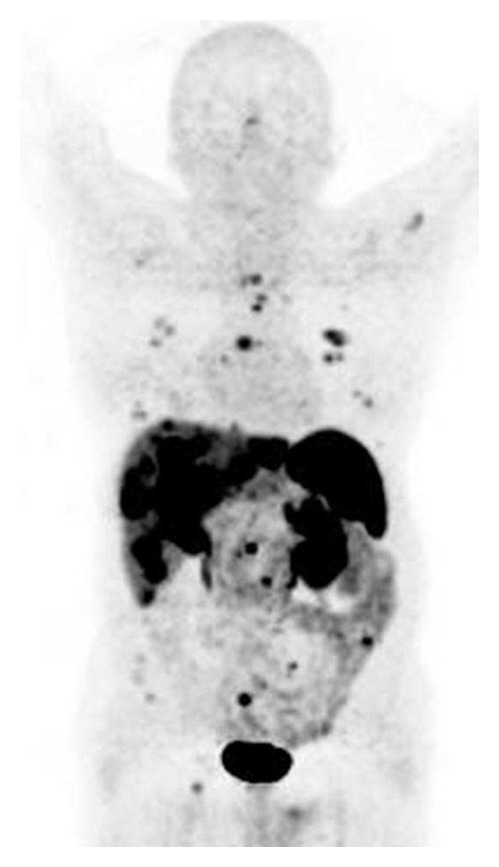
^68^Ga-DOTATOC PET/CT: liver, lung, lymph node, and bone metastases from NET of unknown origin.

Figure 1 shows an example of ^68^Ga-DOTATOC accumulation in tumor lesions. ^68^Ga-DOTATOC positive lesions were preferentially localized at liver, lymph nodes, lung, and skeleton.

An end-treatment ^68^Ga-peptide PET/CT was performed in all treated patients about 3–6 months after the last PRRT administration except for 6 patients in which PD was determined on the basis of worsening of clinical conditions.

**Figure 2:**
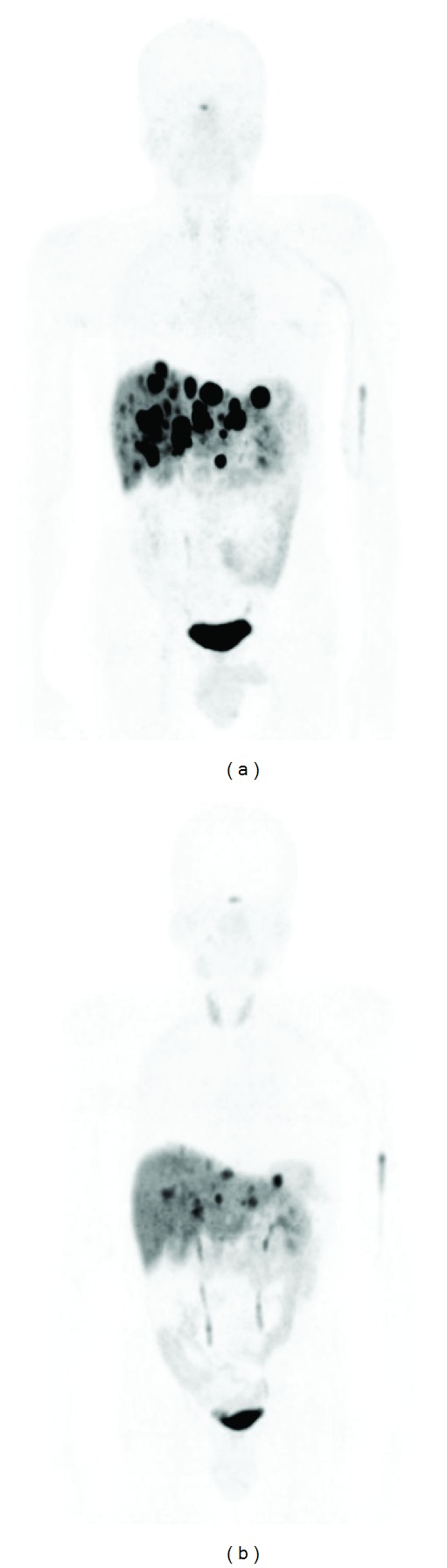
Male, 56 years old, with pancreatic NET and multiple liver metastases. ^68^Ga-DOTATOC PET/CT before therapy (a) and after PRRT (b). The result was a partial response.

**Figure 3:**
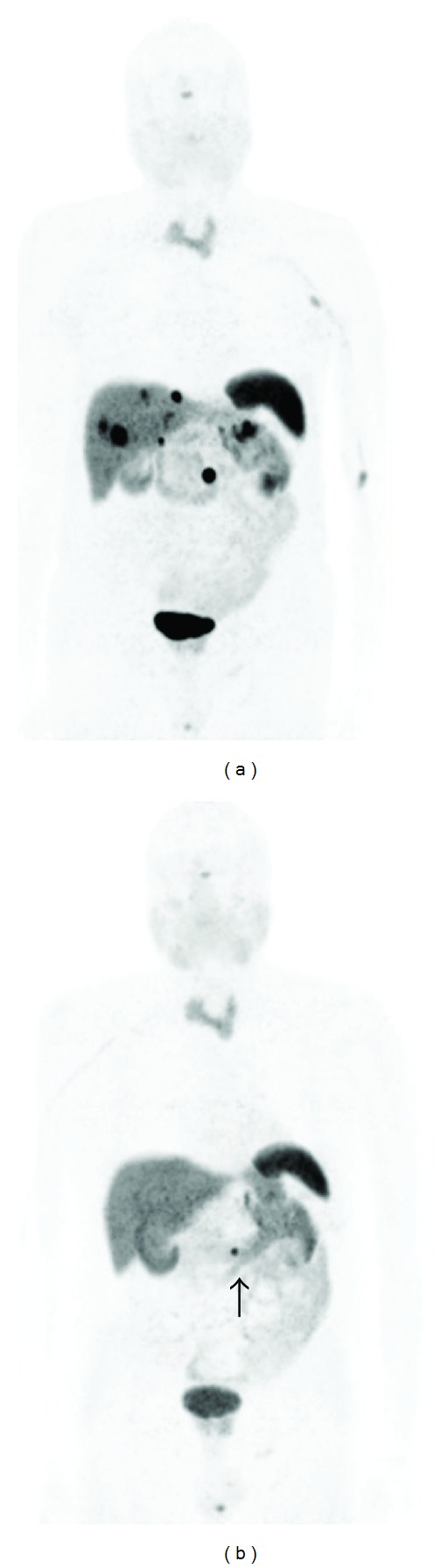
Male, 73 year old, pancreas NET with liver metastases. ^68^Ga-DOTATOC PET/CT before (left) and after therapy (right). PRRT with ^90^Y-DOTATOC (2 cycles) and ^177^Lu-DOTATOC (4 cycles) was administered at interval of 2 months. The response was complete in the liver but partial in the pancreatics region (arrow).

Figures 2 and 3 represent examples of pre- and posttherapeutic ^68^Ga-DOTATOC PET/CT. 

Dosimetric estimates for kidney and bone marrow are summarized in Table 3. No toxicities were recorded after radiopharmaceutical injection administered for dosimetric purpose.

PRRT cycles were administered at 70 ± 24.6 days apart (range 35–140) with a median cumulative activity of 5.5 GBq (range 3.6–7.4 GBq). Thirty-five patients received 4 or 5 PRRT cycles, 10/59 more than 5 cycles while 14/59 patients had <4 PRRT cycles. ^90^Y-PRRT was administered in 33/59 patients (56%), ^177^Lu-PRRT in 9/59 patients (15%) while 17/59 patients (29%) received both ^90^Y-PRRT and ^177^Lu-PRRT in different cycles. Posttherapeutic scintigraphy confirmed a correct distribution of the radiopharmaceutical in all patients.

In 18/59 (30%) patients no adverse effects after administration of the radiopharmaceuticals were observed. Hematological toxicity including grade 2-3 anemia, thrombocytopenia and leukopenia occur in 12/59 patients (20%). Two of the 12 patients who had hematological toxicity presented baseline grade 1 anemia and thrombocytopenia resulting from previous chemotherapies. Asthenia (grade 2-3, 28/59) nausea (grade 1-2, 14/59), vomiting (grade 2-3, 5/59), were frequently observed. Stomatitis (grade 2) and gastritis (grade 1) were also reported in 1 case each. Long-term nephrotoxicity was observed in 3 patients (2 moderate; 1 severe requiring dialysis). Patients who developed nephrotoxicity were treated with ^90^Y-PRRT receiving 5 (2/3) and 6 (1/3) cycles. One of them suffered from both diabetes and blood hypertension. Patient who required dialysis had only one kidney. Clinical benefit was recorded in 21/59 patients while a worsening of clinical conditions was observed in 9/59 patients. All patients which were asymptomatic at baseline and not present modifications of their clinical conditions. 

**Figure 4:**
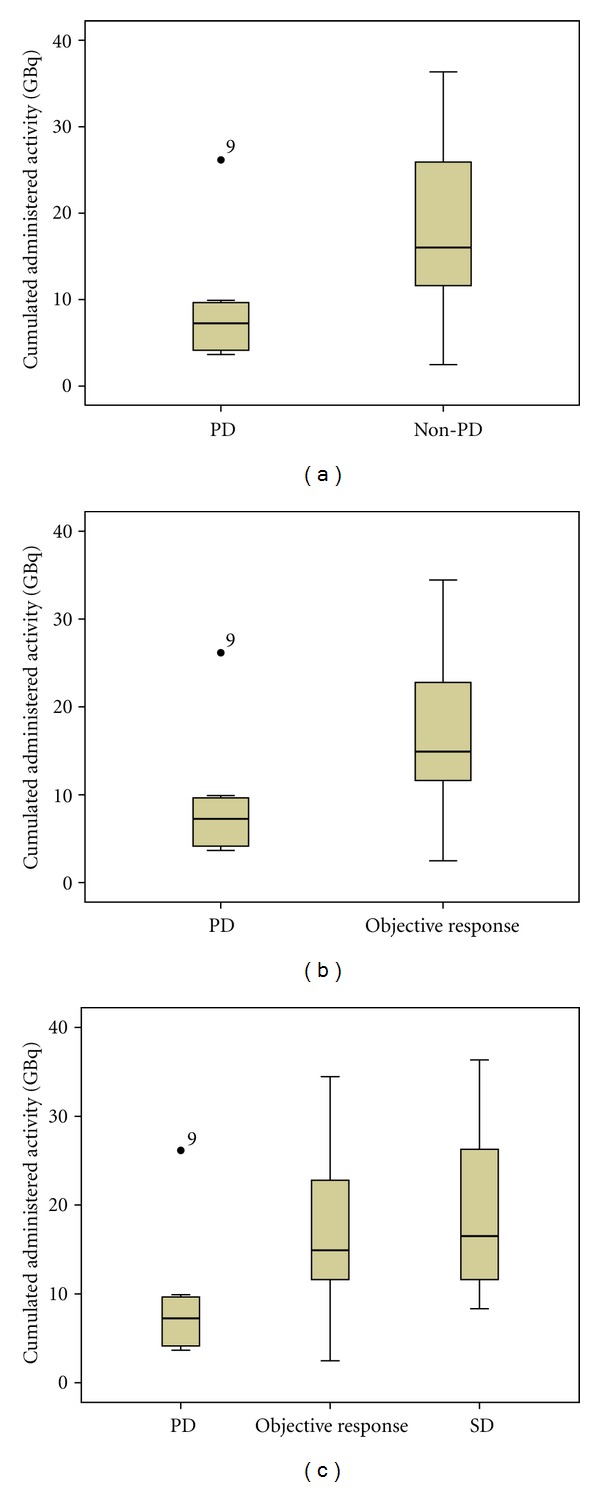
(a) Mann-Whitney test plot shows a significant difference in cumulated administered activity between PD and non-PD patients (*P* < 0.001). Similar results (b) have been obtained by using Mann-Whitney test excluding patients who had SD and thus considering only patients who had objective response (*P* = 0.002). When all subgroups of patients are considered (c), the cumulated administered activity remains significantly different in PD group *versus *both objective response and SD groups while no significant differences have been found in cumulated administered activity comparing patients who presented objective response and stable disease (*P* = 0.435). PD = patients who presented progressive disease; non-PD = patients who presented complete or partial response or disease stabilization; objective response = patients who presented complete or partial response; SD = patients who presented stable disease.

Best objective response was CR in 1/59 patient (2%), PR in 24/59 (40.5%), SD in 24/59 patients (40.5%) while PD was demonstrated in 10/59 (17%) of patients. The overall tumor response rate considering both CR and PR was 42.5%. SUVmax values in the main lesion for both baseline and end-treatment ^68^Ga-peptide PET/CT scans were reported in Table 4 based on functional response. A significant difference in cumulated administered activity between PD and non-PD patients was found as shown by Figure 4. 

Table 5 shows treatment responses based on primary tumor site.  Table 6 shows results of treatment responses based on the type of treatment (^90^Y-PRRT, ^177^Lu-PRRT, or combined ^90^Y-PRRT and ^177^Lu-PRRT) while treatment responses based on the numbers of PRRT cycles are reported in Table 7. In Table 8 functional response was tabulated on the basis of clinical benefit assessment. In the eleven patients with both normal baseline and end-treatment serum CgA levels functional response assessed by ^68^Ga-peptide PET/CT resulted in 1/11 CR, 5/11 PR, 4/11 SD, and 1/11 PD. Discordant results between serum CgA levels trend over time and ^68^Ga-peptide results were found in 23/59 patients. Despite the increase of CgA values ^68^Ga-peptide PET/CT documented a PR in 7 patients and a SD in 6 cases, respectively (Table 9). In one patient classified as SD by ^68^Ga-peptide PET/CT, serum CgA levels completely normalized after PRRT. 

## 4. Discussion

The development of imaging agents specifically designed to target tumor metabolic pathways and associated antigens including membrane receptors opens new horizon both for the selection of patients candidate to target treatment by the *in vivo* detection of enhanced target expressions as well as for the development of new multimodality treatment strategies. 

The expression of SSTRs by NETs made molecular imaging with specific SST-analogues for specific SSTR subtypes the method of choice for their diagnostic workup. In fact ^111^In-labeled SST analogues scintigraphy and more recently ^68^Ga-DOTA-peptides significantly change the diagnostic approach to neuroendocrine tumors. In our study, ^68^Ga-peptide PET/CT as first-selection procedure to determine the presence of high SSTR expression and a tumor uptake at least two times higher than the liver were considered the criteria to be eligible for dosimetric evaluation with ^111^In-peptide. Originally, the study was designed for radiolabelled DOTATOC but because of problem of commercial availability we were obliged to amend the study protocol and substitute DOTATOC with DOTATATE. Using ^68^Ga-peptide PET/CT high SSTR expression to be eligible for subsequent PRRT was found in our study in all patients. Dosimetric estimates confirmed the eligibility of all patients and demonstrated that a fractionated treatment protocol with the intravenous administration of an average activity of 2.6 GBq/cycle for ^90^Y-PRRT and 6.0 GBq/cycle for ^177^Lu-PRRT, respectively, with an interval about 2 months was within the safety threshold of toxicity, particularly for the kidney (the critical organ for PRRT) and the bone marrow. Initially for PRRT, we used ^90^Y since in our department ^177^Lu was allowed from 2009. Subsequently, the criteria of choice of radionuclide used for PRRT was mainly based on tumor size [23] (reserving ^90^Y for lesion(s) >2 cm, ^177^Lu for lesion(s) <2 cm, and ^90^Y/^177^Lu in presence of both conditions) and on dosimetric estimates. 

Administration of ^90^Y-PRRT (average activity of about 2.6 GBq/cycle) and ^177^Lu-PRRT (average activity of about 6.0 GBq/cycle) induced disease control in 83% of patients (1 CR, 24 PR, and 24 SD) with a duration of response of at least 6 months. In the majority of cases, objective response was associated to symptomatic response with an improvement of quality of life. 

These responses rates are comparable with data from literature [11, 24, 25] demonstrating radiological response of 34.1% and clinical response in 29.7% for ^90^Y-PRRT with longer median survival in responders compared to nonresponders (44.7 versus 18.3 months) and response rates of up to 30% with median time to progression of 40 months for ^177^Lu-PRRT [26]. Interestingly, in these studies the degree of uptake on the pretreatment ^111^In-peptide was found to be predictive of response to treatment and overall survival. Despite the small number of patients, combined treatments with labeled peptide using both ^90^Y and ^177^Lu seem to perform better (no evidence of PD) when compared to “single radionuclide” PRRT (8 and 2 cases of PD administering only ^90^Y-peptide and ^177^Lu-peptide, resp.). In our patients population, the SUVmax value in tha main lesion at baseline ^68^Ga-peptide PET/CT examination compared to end-treatment scan was in line with the functional response evaluation. Additionally, in our series of patients, cumulated administered activity was significantly different in responders and no-responders. In agreement with previous reports in literature [25] our data supported the hypothesis that progression at baseline could be a prognostic factor of objective response to PRRT. Indeed previous reports showed also that both SD and objective response (CR+PR) in previously progressive patients showed the same favorable trend [25]. Finally, PRRT showed beneficial effect on symptoms in the majority of patients (36%) and all asymptomatic patients (46%) remained stable over the time. In 39% of our patients discordant results between serum CgA trend and ^68^Ga-peptide findings were observed. Particularly, CgA values increased (with variation up to +263%) in 68% of patients in which PRRT determined PR or SD. Our results on CgA trend and antitumor activity are in contrast with previous reported ones in the literature [25]. However, the majority of our patients assumed proton-pump inhibitor as prophylactic therapy and as well known these drugs may cause substantial increase of blood CgA levels [27] underling the need of a more reliable biomarker to monitoring NETs [28]. 

The prevalence of partial responses and stable disease obtained is mainly related to the advanced stage of the disease when patients are referred to PRRT. In fact, the majority of patients have entered the protocol after the failure of multiple types of treatment situation when more affords are needed to avoid toxicity (i.e., accurate dosimetric evaluation). In this setting of patients an individualized dosimetry has been demonstrated as the ideal method to plan PRRT in order to combine the highest possible dose of radiation to the tumor with the maximum tolerated dose by the dose-limiting organ [29]. In fact, since the absorbed dose to the kidneys and bone marrow might vary considerably between patients, using fixed dose regime side effects may be reduced but at the cost of undertreatment for certain patients, thus, reducing the potential effectiveness of the PRRT. 

The fractionated schedule that we apply in the protocol was selected based on literature data and personal experience. Treatment with this schedule was generally well tolerated with the most common side effects of nausea and vomiting being caused by the administration of amino acid solutions. Reversible bone marrow suppression was seen in 20% of patients. However, in 3 patients we also observed delayed nephrotoxicity requiring in one case dialysis. According to literature data [30] we observed nephrotoxicity in case of ^90^Y-PRRT; however, the presence of high-risk comorbidities in 2/3 cases (only one kidney and blood hypertension plus diabetes each) is not negligible especially if we consider that a low BED threshold (<28 Gy) have been maintained in these specific patients. In all these patients PRRT was administered before the availability of Lu-177. No myelodysplastic syndrome or acute leukemia occurred. 

Despite these interesting results, our study presents some limitations. First the choice of radionuclide used for PRRT was based in some cases on radiopharmaceutical availability at our center. Secondly we evaluate only the functional response. The reason for this choice lies in the fact that the majority of patients (42/59) had liver lesions and their sizes were difficult to accurately be measured using the CT component of PET/CT images without contrast medium. Finally, as previously described, histopathological features of NETs including grading were not comparable avoiding the possibility to further speculate on results. 

## 5. Conclusions

Our study demonstrated that PRRT using a fractionated treatment protocol with the intravenous administration of an average activity of 2.6 GBq/cycle for ^90^Y-PRRT and 6.0 GBq/cycle for ^177^Lu-PRRT, respectively, and with an interval of about 2 months is a feasible therapeutic option for patients with neuroendocrine tumors able to induce disease control in up to 83% of patients, associated with significant clinical response. The use of ^68^Ga-peptide PET/CT as first-selection procedure to determine the presence of high SSTR expression followed by standard dosimetric estimates may be used for patients selection. 

However, there is the clinical need of randomized clinical trials to determine what is optimal treatment schedule based on the specific biological and molecular features of the tumor. Future therapeutic trials should also aim to include patients at earlier stage of disease and to investigate the best setting where to introduce radioreceptor therapy in combination, rather than an alternative, to other treatment options.

## Figures and Tables

**Table 1:** Site and number of metastasis in the 59 evaluated patients at the enrollment in the clinical trial.

Site of metastasis	Number of metastasis	
	≤5	>5
Bone (21/59)	0/21	21/21
Liver (42/59)	2/42	40/42
Lung (4/59)	0/4	4/4
Lymph nodes (34/59)	3/34	31/34
Other (6/59)	1/6	5/6

**Table 2:** List of previous treatments in the 59 evaluated patients order on the basis of their frequency.

Previous treatment	Number of patients
Surgery	39/59
“Cold” SST analogues	25/59
Chemotherapy	13/59
TACE or RFTA	7/59
External beam radiotherapy	5/59

TACE: intra-arterial hepatic chemoembolization; RFTA: radiofrequency thermoablation.

**Table 3:** Dosimetric estimates for kidney and bone marrow.

	Mean	Median	SD	Range
^ 90^Y-kidney dose (Gy/GBq)	2.4*E* + 00	1.5*E* + 00	1.9	0.32–8.90
^ 90^Y-bone marrow dose (Gy/GBq)	9.7*E* − 02	5.8*E* − 02	0.1	0.0047–0.51
^ 177^Lu-kidney dose (Gy/GBq)	3.9*E* − 01	2.5*E* − 01	0.3	0.05–1.47
^ 177^Lu-bone marrow dose (Gy/GBq)	2.81*E* − 02	1.29*E* − 02	0.04	0.0163–0.256

**Table 4:** Baseline and end-treatment SUV max values recorder for all patients in the main lesion assessed by  ^68^Ga-peptide PET/CT tabulated on the basis of patients' functional response.

	Main lesion					
	Baseline SUV max value			End-treatment SUV max value		
Functional response	Mean	Median	Range	Mean	Median	Range
CR	45.3	45.3	—	0.9	0.9	—
PR	79.7	29.2	6.3–119.9	39.2	17.8	3.2–49.1
SD	31.1	20.9	6.6–82.0	31.2	28.2	11.2–61.3
PD^ç^	27.9	22	11.7–55.8	35.7	26	15.7–74.7

^ç^Data from the 4 patients in which PD was assessed using  ^68^Ga-peptide PET/CT.

**Table 5:** Results of treatment responses tabulated on the basis of primary tumor site.

Site of primitive tumor	CR	PR	SD	PD
GI (19/59)	—	8/19 (42%)	9/19 (47%)	2/19 (11%)
Pancreas (16/19)	1/16 (6%)	5/16 (31%)	6/16 (38%)	4/16 (25%)
Lung (13/59)	—	8/13 (62%)	3/13 (23%)	2/13 (15%)
Unknown origin (11/59)	—	3/11 (27%)	6/11 (55%)	2/11 (18%)

**Table 6:** Results of treatment responses tabulated on the basis of the type of treatment.

Type of treatment	CR	PR	SD	PD
^ 90^Y-PRRT (33/59)	1/33 (3%)	13/33 (40%)	11/33 (33%)	8/33 (24%)
^ 177^Lu-PRRT (10/59)	—	2/10 (20%)	6/10 (60%)	2/10 (20%)
Both ^90^Y-PRRT and ^177^Lu-PRRT (16/59)	—	9/16 (56%)	7/16 (44%)	—

**Table 7:** Results of treatment responses tabulated on the basis of numbers of PRRT cycles.

Numbers of cycles	CR	PR	SD	PD^#^
2-3 (11/59)	1/11 (9%)	5/11 (46%)	2/11 (18%)	3/11 (27%)
4-5 (35/59)	—	14/35 (40%)	17/35 (49%)	4/35 (11%)
>5 (10/59)	—	5/10 (50%)	5/10 (50%)	—

^#^Three patients were treated with only one cycle.

**Table 8:** Results of functional response to PRRT assessed by  ^68^Ga-peptide PET/CT based on clinical benefit evaluation.

	Functional response			
Clinical response	CR	PR	SD	PD
Clinical benefit	—	5	16	—
SD	—	—	1	1
PD	—	—	—	9
Asymptomatic patients	1	19	7	—

**Table 9:** Trend of serum CgA values comparing baseline and end-treatment levels are tabulated on the basis of patients' functional response in the 23/59 cases in which discordant results between CgA trend and ^68^Ga-peptide PET/CT results were observed.

Functional response	CgA values		
	Increased	Stable^$^	Decreased
PR (9/23)	7^∗^	2	—
SD (10/23)	6^∧^	—	4^§^
PD (4/23)	—	4	—

^$^Variation  ≤ 10%;  ^∗^mean variation = 271 ± 220 (range 15–682); ^∧^mean variation = 104 ± 119 (range 3–305); ^§^mean variation = 669 ± 554 (range 41–1343).

## References

[1] Yao JC, Hassan M, Phan A (2008). One hundred years after “carcinoid”: epidemiology of and prognostic factors for neuroendocrine tumors in 35,825 cases in the United States. *Journal of Clinical Oncology*.

[2] Bodei L, Ferone D, Grana CM (2009). Peptide receptor therapies in neuroendocrine tumors. *Journal of Endocrinological Investigation*.

[3] Rindi G, Klöppel G, Alhman H (2006). TNM staging of foregut (neuro)endocrine tumors: a consensus proposal including a grading system. *Virchows Archiv*.

[4] Kulke MH, Siu LL, Tepper JE (2011). Future directions in the treatment of neuroendocrine tumors: consensus report of the National Cancer Institute Neuroendocrine Tumor clinical trials planning meeting. *Journal of Clinical Oncology*.

[5] Kaltsas GA, Rockall A, Papadogias D, Reznek R, Grossman AB (2004). Recent advances in radiological and radionuclide imaging and therapy of neuroendocrine tumours. *European Journal of Endocrinology*.

[6] Versari A, Camellini L, Carlinfante G (2010). Ga-68 dOTATOC PET, endoscopic ultrasonography, and multidetector CT in the diagnosis of duodenopancreatic neuroendocrine tumors: a single-centre retrospective study. *Clinical Nuclear Medicine*.

[7] Baum RP, Virgolini I, Ambrosini V (2010). Procedure guidelines for PET/CT tumour imaging with ^68^Ga-DOTA-conjugated peptides: ^68^Ga-DOTA-TOC, ^68^Ga-DOTA-NOC, ^68^Ga-DOTA-TATE. *European Journal of Nuclear Medicine and Molecular Imaging*.

[8] Naswa N, Sharma P, Kumar A (2011). Gallium-68-DOTA-NOC PET/CT of patients with gastroenteropancreatic neuroendocrine tumors: a prospective single-center study. *American Journal of Roentgenology*.

[9] de Jong M, Bernard BF, De Bruin E (1998). Internalization of radiolabelled [DTPA^0^]octreotide and [DOTA^0^,Tyr^3^]octreotide: peptides for somatostatin receptor-targeted scintigraphy and radionuclide therapy. *Nuclear Medicine Communications*.

[10] Slooter GD, Breeman WA, Marquet RL (1999). Anti-proliferative effect of radiolabeled octreotide in a metastases model in rat liver. *International Journal of Cancer*.

[11] Virgolini IJ, Gabriel M, von Guggenberg E, Putzer D, Kendler D, Decristoforo C (2009). Role of radiopharmaceuticals in the diagnosis and treatment of neuroendocrine tumours. *European Journal of Cancer*.

[12] Bombardieri E, Virgolini I (2010). Diagnosis and therapy of neuroendocrine tumours. *Quarterly Journal of Nuclear Medicine and Molecular Imaging*.

[13] Kunikowska J, Królicki L, Hubalewska-Dydejczyk A, Mikołajczak R, Sowa-Staszczak A, Pawlak D (2011). Clinical results of radionuclide therapy of neuroendocrine tumours with ^90^Y-DOTATATE and tandem ^90^Y/^177^Lu-DOTATATE: which is a better therapy option?. *European Journal of Nuclear Medicine and Molecular Imaging*.

[14] Ambrosini V, Fani M, Fanti S (2011). Radiopeptide imaging and therapy in Europe. *Journal of Nuclear Medicine*.

[15] Grassi E, Sghedoni R, Asti M, Fioroni F, Salvo D, Borasi G (2009). Radiation protection in 90Y-labelled DOTA-D-Phe1-Tyr3-octreotide preparations. *Nuclear Medicine Communications*.

[16] Wehrmann C, Senftleben S, Zachert C, Müller D, Baum RP (2007). Results of individual patient dosimetry in peptide receptor radionuclide therapy with ^177^Lu DOTA-TATE and ^177^Lu DOTA-NOC. *Cancer Biotherapy and Radiopharmaceuticals*.

[17] Breeman WA, De Jong MT, De Blois E, Bernard BF, De Jong M, Krenning EP (2004). Reduction of skeletal accumulation of radioactivity by co-injection of DTPA in [^90^Y-DOTA^0^,Tyr^3^]octreotide solutions containing free ^90^Y^3+^. *Nuclear Medicine and Biology*.

[18] Decristoforo C, Knopp R, von Guggenberg E (2007). A fully automated synthesis for the preparation of 68Ga-labelled peptides. *Nuclear Medicine Communications*.

[19] Asti M, De Pietri G, Fraternali A (2008). Validation of ^68^Ge/^68^Ga generator processing by chemical purification for routine clinical application of ^68^Ga-DOTATOC. *Nuclear Medicine and Biology*.

[20] Stabin MG, Sparks RB, Crowe E (2005). OLINDA/EXM: the second-generation personal computer software for internal dose assessment in nuclear medicine. *Journal of Nuclear Medicine*.

[21] Cremonesi M, Ferrari M, Zoboli S (1999). Biokinetics and dosimetry in patients administered with ^111^In-DOTA-Tyr^3^-octreotide: implications for internal radiotherapy with ^90^Y-DOTATOC. *European Journal of Nuclear Medicine*.

[22] National Cancer Institute (2006). *CTEP: NCI Guidance on CTC Terminology Applications*.

[23] de Jong M, Breeman WA, Valkema R, Bernard BF, Krenning EP (2005). Combination radionuclide therapy using ^177^Lu and ^90^Y-labeled somatostatin analogs. *Journal of Nuclear Medicine*.

[24] Gabriel M, Andergassen U, Putzer D (2010). Individualized peptide-related-radionuclide-therapy concept using different radiolabelled somatostatin analogs in advanced cancer patients. *Quarterly Journal of Nuclear Medicine and Molecular Imaging*.

[25] Bodei L, Cremonesi M, Grana CM (2011). Peptide receptor radionuclide therapy with ^177^Lu-DOTATATE: the IEO phase I-II study. *European Journal of Nuclear Medicine and Molecular Imaging*.

[26] Kwekkeboom DJ, de Herder WW, Kam BL (2008). Treatment with the radiolabeled somatostatin analog [^177^Lu- DOTA0,Tyr^3^]octreotate: toxicity, efficacy, and survival. *Journal of Clinical Oncology*.

[27] Glinicki P, Jeske W (2010). Chromogranin A (CgA)—the influence of various factors in vivo and in vitro, and existing disorders on it’s concentration in blood. *Endokrynologia Polska*.

[28] Ito T, Igarashi H, Jensen RT (2012). Serum pancreastatin: the long sought universal, sensitive, specific tumor marker for neuroendocrine tumors?. *Pancreas*.

[29] Bodei L, Cremonesi M, Ferrari M (2008). Long-term evaluation of renal toxicity after peptide receptor radionuclide therapy with ^90^Y-DOTATOC and ^177^Lu-DOTATATE: the role of associated risk factors. *European Journal of Nuclear Medicine and Molecular Imaging*.

[30] Barone R, Borson-Chazot F, Valkema R (2005). Patient-specific dosimetry in predicting renal toxicity with ^90^Y-DOTATOC: relevance of kidney volume and dose rate in finding a dose-effect relationship. *Journal of Nuclear Medicine*.

